# The vaginal microbial communities of healthy expectant Brazilian mothers and its correlation with the newborn’s gut colonization

**DOI:** 10.1007/s11274-019-2737-3

**Published:** 2019-10-10

**Authors:** Priscila Dobbler, Volker Mai, Renato S. Procianoy, Rita C. Silveira, Andréa L. Corso, Luiz Fernando Wurdig Roesch

**Affiliations:** 1 Centro Interdisciplinar de Pesquisas em Biotecnologia – CIP-Biotec, Campus São Gabriel, Universidade Federal do Pampa, São Gabriel, Rio Grande do Sul Brazil; 2 Department of Epidemiology, College of Public Health and Health Professions and College of Medicine, Emerging Pathogens Institute, University of Florida, Gainesville, FL 32611 USA; 3 Serviço de Neonatologia do Hospital de Clínicas de Porto Alegre, Universidade Federal do Rio Grande do Sul, Porto Alegre, Rio Grande do Sul Brazil

**Keywords:** 16S rRNA, Microbial diversity, Next generation sequencing, Pregnancy, Vaginal microbiome

## Abstract

The female lower genital tract harbors a complex microbial community essential for homeostasis and health. During pregnancy, the female body undergoes unique hormonal changes that contribute to weight gain as well as modulations in immune function that can affect microbiota composition. Several studies have described the vaginal microbiota of pregnant women from the USA, Europe and Mexico. Here we expand our knowledge about the vaginal microbial communities during the third trimester to healthy expectant Brazilian mothers. Vaginal samples were collected from patients delivering at the Hospital de Clínicas de Porto Alegre, Brazil. Microbial DNA was isolated from samples and the V4 region of the 16S rRNA gene was amplified and sequenced using the PGM Ion Torrent. Brazilian pregnant women presented three distinct types of microbial community at the time of labor. Two microbial communities, Cluster 1 and Cluster 3, presented an overall dominance of *Lactobacillus* while Cluster 2 tended to present higher diversity and richness, with the presence of *Pseudomonas*, *Prevotella* and other vaginosis related bacteria. About half of the Brazilian mothers sampled here had dominance of *L. iners*. The proportion of mothers without dominance of any *Lactobacillus* was higher in Brazil (22%) compared to UK (2.4%) and USA, where this community type was not detected. The vaginal microbiota showed significant correlation with the composition of the babies’ gut microbiota (p-value = 0.002 with a R^2^ of 15.8%). Mothers presenting different vaginal microbiota shared different microorganisms with their newborns, which would reflect on initial colonizers of the developing newborns’ gut.

**Electronic supplementary material:**

The online version of this article (10.1007/s11274-019-2737-3) contains supplementary material, which is available to authorized users.

## Introduction

The female lower genital tract harbors a complex microbial community essential for homeostasis and health. Although complex in terms of microbial composition, the healthy vaginal microbiota, in non-pregnant woman, is dominated by *Lactobacillus* species. *Lactobacillus* spp. produce lactic acid as their main fermentation product which contributes to maintaining a healthy vaginal environment by antimicrobial effect associated with a reduced pH (Boskey et al. 1999; Tachedjian et al. 2017). Newborn’s health outcome is another important role of a healthy vaginal microbiota. Infant’s gut microbial community is shaped also during birth, trough the birth canal, influencing the initial gut microbial community assembly (Dominguez-Bello et al. 2010; Milani et al. 2017).

During pregnancy, the female body undergoes hormonal changes contributing to weight gain as well as modulations in immune function that could be associated with changes in mothers’ microbiota composition (Nuriel-Ohayon et al. 2016). In contrast to various disease states, where microbiota alterations correlate with adverse outcomes, microbiota changes during pregnancy might contribute to a healthy full term pregnancy. Throughout the first trimester, the relative abundance of *Lactobacillus* spp. increases while the abundance of other anaerobic bacteria such as *Sneathia*, *Gardnerella*, *Parvimonas, Gemella* and *Dialister* decreases. Towards the last trimester the vaginal microbiota stabilizes but with lower diversity compared to non-pregnant woman (Romero et al. 2014a, b).

The presence of *Lactobacillus* spp. as a member of the healthy vaginal microbiota seems to occur irrespective of geography or racial background, though varying in overall abundance and prevalence. One of the questions this study seeks to answer is if this would also be found on a Brazilian cohort. Several studies have described the vaginal microbiota of pregnant women from the USA, Europe or Mexico (Hernández-Rodríguez et al. 2011; Hyman et al. 2014; MacIntyre et al. 2015; Romero et al. 2014a, b). Romero et al. (2014b) compared the vaginal microbiota of non-pregnant (N = 32), 50% African American (AA) with pregnant women (N = 22, 86% AA) and monitored microbiota changes throughout a term pregnancy. In another study Romero et al. (2014a) investigated microbiota differences between term (N = 72, 86% AA) and preterm (N = 18, 94% AA) delivery. Hernández-Rodríguez et al. (2011) described the vaginal microbiota during the third trimester of gestation in 23 pregnant Mexican women. MacIntyre et al. (2015) found a higher proportion of women with dominance of *L. jensenii* in the UK compared to women from USA. Bisanz et al. (2015) also described the vaginal microbiota of 56 pregnant women (53 with term gestation) in a rural region of Tanzania, though there were no description of types of vaginal communities. They found that the majority of the women sampled had dominance of *Lactobacillus* spp. (no species resolution). *Prevotella*, *Gardnerella*, *Sneathia* were also found in lower proportions.

The maternal vaginal microbiota contributes to the colonization of the newborn’s gut. Initial infant’s gut colonization is very important for early and long-term health. Initial abnormal microbial transfer can affect the immune system development, allergy and asthma future incidence (Johnson and Ownby 2017; Milani et al. 2017), and can contribute to postnatal complications including early onset sepsis (Madan et al. 2012; Wortham et al. 2016). Women with distinct vaginal microbial communities during labor onset might transfer different microbial seeds to their newborn’s gut. Thus, understanding how these different vaginal microbial communities are presented during labor, could provide an avenue for developing microbiota-targeting interventions that can improve maternal and newborn’s health.

The purpose of this study was to characterize the vaginal microbial community of healthy pregnant Brazilian women at the end of their third trimester, and understand how it correlates with their respective infant’s gut microbiota colonization at time of birth. To our knowledge, there are no reports on how the vaginal microbiota of healthy pregnant women from Brazil.

## Materials and methods

We performed an observational, cross-sectional study based on a convenience sampling strategy. Participants were recruited at the Neonatology Section of Hospital de Clínicas de Porto Alegre (HCPA), Brazil, between the years of 2014 and 2015. Expectant mothers were enrolled at hospital admission for delivery and provided written informed consent. The study protocol was approved by the Ethics Committee of Hospital de Clínicas de Porto Alegre (HCPA), approval number 39164114.0.0000.532. Exclusion criteria: (1) HIV carrier, (2) recreational drug user or alcohol dependent (self-reported), (3) urinary tract infections, (4) any antibiotic usage during third trimester, (5) gestational diabetes and (6) congenital infections in newborn. We obtained samples from a total of 45 pregnant women delivering at 37–40 weeks of gestational age and 45 first fecal samples (meconium) from their babies. Samples from 18 women were excluded from the analysis based on: collection after delivery (n = 1), lack of records for collection time (n = 8), urinary tract infection in the third trimester (n = 2), intrapartum antibiotic treatment (n = 2), gestational diabetes (n = 2), and low sequence coverage, with less than 1000 sequences (n = 3). Thus, vaginal samples from 27 expecting mothers were retained for this analysis, and 26 samples of first pass meconium from their respective newborns. All babies, except one, were vaginally delivered. Vaginal samples were collected after hospital admission and shortly before delivery by rotating a sterilized swab five times along the vaginal lumen with a circular motion. Speculum was not used. There were no occurrences of Premature Rupture of Membranes (PROM) or administration of intravenous antibiotics during delivery. Meconium samples were collected within 24 h of birth from a single diaper directly into a sterile collection tube. All samples were immediately stored at − 80 °C for later analysis.

### Microbial DNA extraction, 16S rRNA amplification and library preparation

Microbial DNA isolation from vaginal and meconium samples, amplification of the 16S rRNA, and sequencing protocol were performed following Dobbler et al. (2017, 2018). Raw sequences were deposited in the Sequence Read Archive (SRA), accession SRP093885. Records are accessible at https://www.ncbi.nlm.nih.gov/sra/SRP093885. Run numbers SRR7657414 to SRR7657440.

### Sequence processing and statistical analysis

The 16S rRNA raw sequences were analyzed following the recommendations of the Brazilian Microbiome Project (Pylro et al. 2014) and as previously described (Dobbler et al. 2017). For downstream analysis, the data set was filtered by removing Chloroplast/Cyanobacteria sequences and only OTUs with more than 5 sequence reads were kept before rarefying to the same number of sequences (Lemos et al. 2011). Observed OTU richness and Shannon diversity index estimators were calculated using the “phyloseq” package (McMurdie and Holmes 2013), and plotted using the “ggpubr” package, both in the R environment. Alpha diversity measurements were tested for normality with Shapiro–Wilk test, and clusters differences were evaluated with the Kruskall–Wallis test. Clinical data was also evaluated, including testing of quantitative variables for normality with Shapiro–Wilk Normality Test. Quantitative variables with normal distribution were compared by the ANOVA test while the non-normal distributed variables were compared by Kruskal–Wallis rank sum test.

We applied an unsupervised clustering approach on the different vaginal microbial communities occurring in Brazilian expectant mothers. First, a Bray–Curtis dissimilarity matrix was built with the OTUs identified in each sample. A Hopkins statistic test was used to verify cluster tendency, followed by the Gap statistical analysis (Tibshirani et al. 2001) to discover the number of clusters in the dataset. Gap statistic was performed with 500 Monte Carlo simulations. The members of each cluster were then identified using k-means with the number of clusters derived from the previous analysis, with 25 different random starting assignments. Analysis was carried out using the “cluster” and “phyloseq” packages (Maechler 2013; McMurdie and Holmes 2013) implemented in R environment.

To test the hypothesis that different vaginal microbial communities occur in healthy Brazilian mothers, Bray–Curtis dissimilarity matrix was ordinated by Multidimensional Scaling (MDS) and differences among community states were tested by Permutational Multivariate Analysis of Variance (PERMANOVA) (Anderson 2001) implemented in the vegan package (Oksanen et al. 2015), and a pairwise PERMANOVA. Also, in order to identify the main taxa responsible for the differences among each community type, the 30 most abundant OTUs were biploted with the Bray–Curtis dissimilarity matrix in the MDS space and the mean relative abundance were computed for each community type.

High-level phenotype of these microbial communities was investigated through BugBase platform (Ward et al. 2017). For that, the raw 16S rRNA dataset was prepared following the instructions of Langille et al. (2013). After quality filtering and trimming, OTUs were picked against the Greengenes (McDonald et al. 2012) database. Hypothesis testing was performed with Pairwise Mann–Whitney–Wilcoxon Tests.

After exploration of maternal vaginal microbial communities, we also sought to understand whether these different vaginal microbial communities were associated with differences in the newborn’s gut microbial community assembly at birth. To accomplish this, OTUs with more than 5 reads were retained and Bray–Curtis dissimilarity and Binary matrices were constructed and ordinated by MDS, where babies’ samples were grouped according with their respective mother’s cluster. Hypothesis testing was performed with PERMANOVA. Also, in order to visualize how maternal and newborn’s sample are clustered, a heatmap was constructed with taxa present in at least 10% in one sample using the ‘pheatmap’ R package (Kolde 2019).

Metagenomics core exploration tool (MetaCoMET) (Wang et al. 2016) was used to find shared OTUs between mother’s clusters and their newborns. An OTU was considered member of a group when the cumulative relative abundance was above 0.1%.

## Results

### Overall 16S sequencing report and diversity description

After initial quality filtering that retained all OTU’s except singletons, Good’s coverage at 97% similarity cutoff ranged from 89 to 99% of sequencing coverage (Supplementary Table S1). Further analyses were performed after removing OTUs with less than six sequences across all samples. In all, after quality assessment and pruning of low representative OTUs, 745,688 sequences were retained with a median of 7236 sequences per sample.

Alpha diversity of the vaginal microbial communities at 37–40 weeks of gestational age varied greatly among mothers. On average, the number of observed OTUs among the subjects was 26, with a minimum of 9 and a maximum of 345 OTUs. The Shannon diversity index ranged from 0.14 to 5.18 with an average of 1.27. The number of phyla and genera also presented great variation among the mothers ranging from 1 to 15 and 1 to 81 respectively.

Similarly, the newborn’s gut microbiota also presented great variation in alpha diversity. The number of OTUs ranged from 12 to 199 with a mean of 74.7 OTUs per sample. Shannon Diversity Index ranged from 0.4 to 4.1 with a mean of 2.1 per sample.

### Determining whether vaginal microbial communities differ among healthy pregnant mothers

To determine whether the vaginal microbial community of Brazilian pregnant woman represented distinct clusters, we applied an unsupervised machine learning approach (Supplementary Table S2). The first step consisted in verifying the cluster tendency using the Hopkins statistic. The Hopkins statistical analysis of the Bray–Curtis dissimilarity distance matrix at OTU level was 0.19, which indicated the presence of clusters. Gap statistic, using the same dissimilarity matrix ordinated by a Multidimensional Scaling space, identified that Brazilian mothers had three distinct vaginal microbiota clusters. With a Gap statistic value of 0.407 and a standard error of 0.037 (Supplementary Table S2).

The k-means clustering function was applied to determine cluster membership. The analysis was carried out using the number of clusters specified by the Gap statistic and with 25 different random starting cluster assignments. K-means clustering method selects the best assignment of cluster members that produces the lowest within cluster variation. Seven pregnant mothers were assigned to the Cluster 1, six pregnant mothers were assigned to Cluster 2 and fourteen pregnant mothers were assigned to Cluster 3.

### Microbial community analysis among clusters

**Fig. 1:**
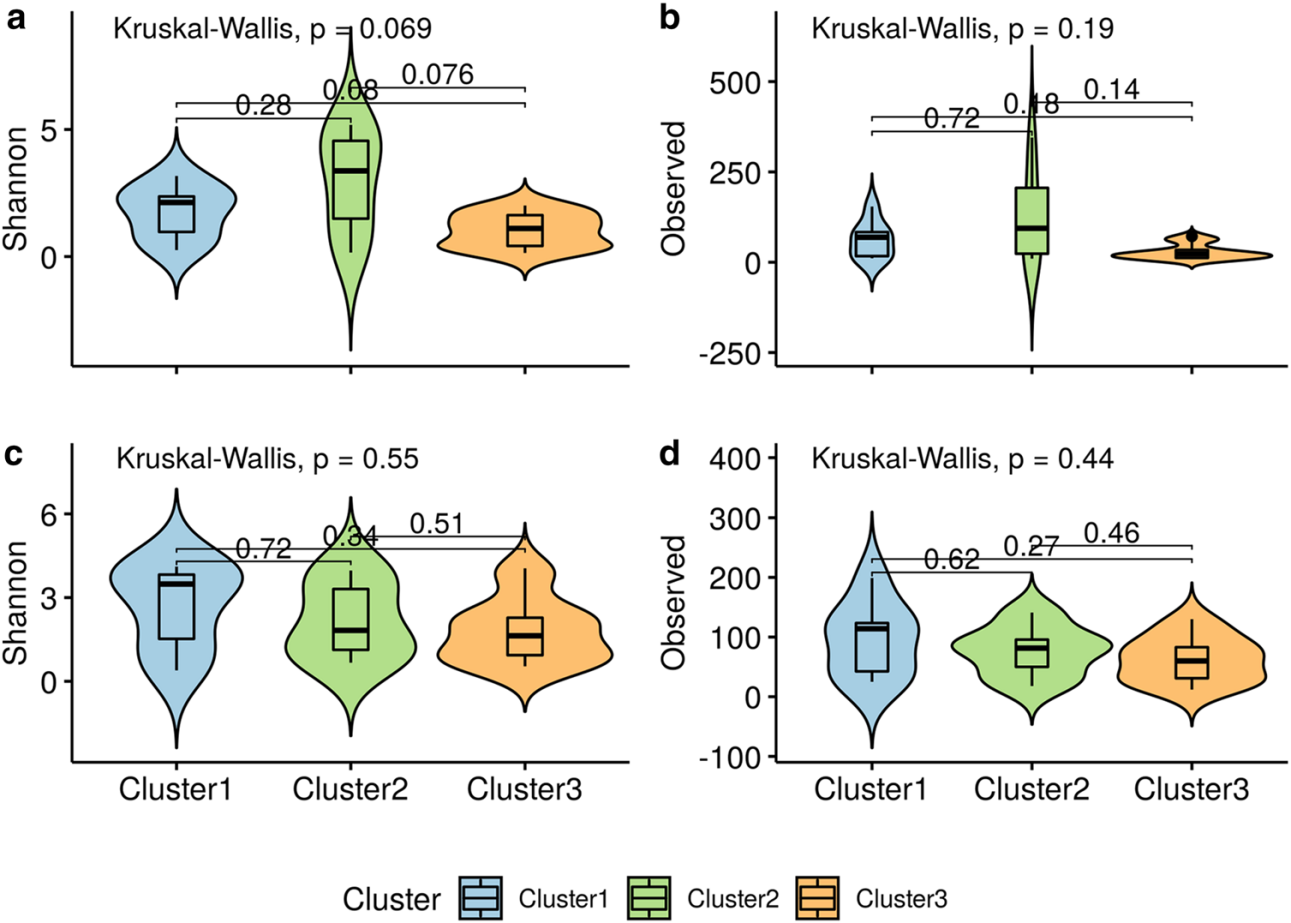
Violin plot representing alpha diversity measurements. **a** Shannon Diversity Index and **b** observed OTUs of maternal vaginal samples. **c** Shannon Diversity Index and **d** observed OTUs of newborns’ meconium samples. Colors indicate cluster classification, babies samples are colored according to mother’s cluster. Observed stands for the number of OTUs found in each cluster and Shannon stands for Shannon Diversity Index. Boxes span the first to third quartiles; the horizontal line inside the boxes represents the median black dots represents all samples in each group and red dots represent outliers

The Shannon Diversity Index and the number of OTUs were used in order to evaluate how alpha diversity compared between the clusters. We found that there was a marginal difference in alpha diversity between the vaginal microbial clusters, global p-value for Shannon Index was 0.069, but no overall difference in number of observed OTUs (p-value = 0.19 (Fig. 1a, b). There was a tendency of higher Shannon diversity in Cluster 2 compared to Cluster 3 (p-value = 0.076). There were no difference in Shannon diversity (Fig. 1c) and number of observed OTUs (Fig. 1d) of the microbial community of the babies’ gut when considering their respective mothers cluster.

**Fig. 2:**
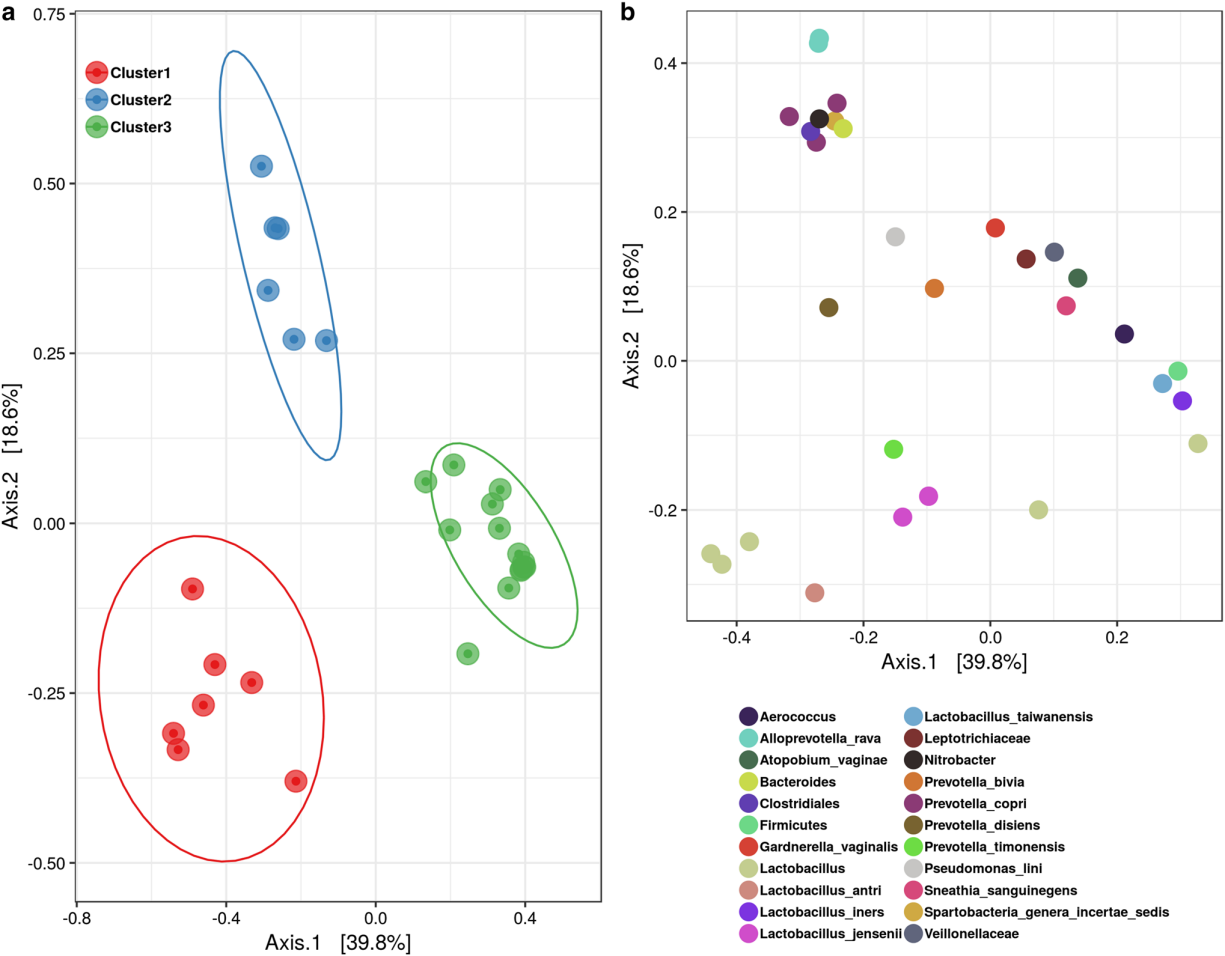
Multidimensional Scaling of the Bray–Curtis distance of vaginal samples. Each symbol represents a microbial community of an individual sample. **a** Presents the different clusters of vaginal microbial communities. Each color represents a cluster, large circles around samples represents a confidence ellipse of 95%. **b** Represents the 30 most abundant OTUs across all vaginal samples, summarized at the highest taxonomy level with at least 80% confidence, into 22 different taxa. Each circle represents a different OTU, while different colors represent different taxonomy assignments and indicate which OTU/taxa are driving sample clustering

In order to understand how the structure of the microbial communities of each vaginal sample compared between groups. We applied the MDS to the Bray–Curtis dissimilarity matrix, as a measure of Beta-diversity, and plotted it with taxa abundance The first two axis alone explained 54.7% of the variation between the microbial communities, and the mothers’ vaginal microbial communities formed three clearly distinct groups (Fig. 2). In addition, a PERMANOVA analysis revealed that the microbial composition of the three community types were different (p-value = 0.001, R^2^ = 0.50), and indicated that 50% of the distance variation was explained by the different microbial communities’ membership (Table 1). All clusters were statistically different (p-adjusted = 0.003) and the largest R^2^ was observed between Cluster 3 and Cluster 1, which explained more than 56% of the difference between these communities (Table 1). The large difference observed among clusters (measured by the R^2^) indicated this contrast present biological relevance. In addition, analysis of multivariate homogeneity of group dispersions suggests that differences between Cluster 1 and Cluster 3 are not caused by differences in homogeneity of variance (Supplementary Table S3).

**Table 1:** Permutational multivariate analysis of variance among the vaginal microbial communities of different community clusters found in this study

	DF	SS	F. Model	R^2^	P-value	P-adjusted
Clusters	2	4.283	12.245	0.50505	0.001	–
Residuals	24	4.197	–	0.49495	–	–
Total	26	8.481	–	1.0	–	–
Pairwise clusters comparisons						
Cluster 3 vs. Cluster 2			10.873101	0.3765824	0.001	0.003*
Cluster 3 vs. Cluster 1			24.232420	0.5605150	0.001	0.003*
Cluster 2 vs. Cluster 1			5.125949	0.3178696	0.001	0.003*

*P-value adjusted for multiple comparisons with Bonferroni

### Clinical features of each vaginal community type and microbial composition

After community state assignment, the clinical characteristics of each group were evaluated. We investigated whether gestational age, maternal age, number of pregnancies or number of prenatal visits differed between community states (Table 2). After testing for data normality, Kruskal–Wallis was applied for non-normal data, and Analysis of Variance were applied for data with normal distribution. We found that there was no significant difference in gestational age (p-value = 0.5), mother’s age (p-value = 0.54), in the number of pregnancies (p-value = 0.25) or number of prenatal visits (p-value = 0.3801) between the community states. Though, our few samples might not have enough power to detect differences.

**Table 2:** Sample group characteristics summarized according to the different vaginal microbial communities found in this study

	Cluster 1 (n = 7)	Cluster 2 (n = 6)	Cluster 3 (n = 14)	p-values
Mothers’ characteristics				
Gestational age (weeks)***	39.77 ± 1.0	39.14 ± 1.1	39.73 ± 1.1	0.5*
Mother’s age (years)	27.14 ± 7.4	24.33 ± 3.6	23.71 ± 6.0	0.3931**
Number of pregnancies	2.3 ± 1.1	2.3 ± 0.5	1.7 ± 0.9	0.2308**
Prenatal visits	6.71 ± 2.14	8.17 ± 1.72	7.14 ± 1.92	0.3801**
Newborns’ characteristics				
Weight at birth (g)	3362.86 ± 414.82	3370.83 ± 279.15	3924.43 ± 270.78	0.838*
Length (cm)	48.92 ± 1.5	48.5 ± 1.61	48.54 ± 0.82	0.782*
APGAR 1	8.29 ± 1.25	8.67 ± 0.52	8.21 ± 2.33	0.7581**
APGAR 5	9.14 ± 0.69	9.67 ± 0.52	9.29 ± 0.83	0.3412**
Head circumference (cm)	33.17 ± 1.51	33.5 ± 1	34.14 ± 1.51	0.337*
Thoracic circumference (cm)	33.67 ± 1.37	33.58 ± 1.24	33.54 ± 1.25	0.978*

Values expressed in means and standard deviations of the mean

*ANOVA

**Kruskal–Wallis rank sum test

***Gestational age for delivery time and sample collection time

**Fig. 3:**
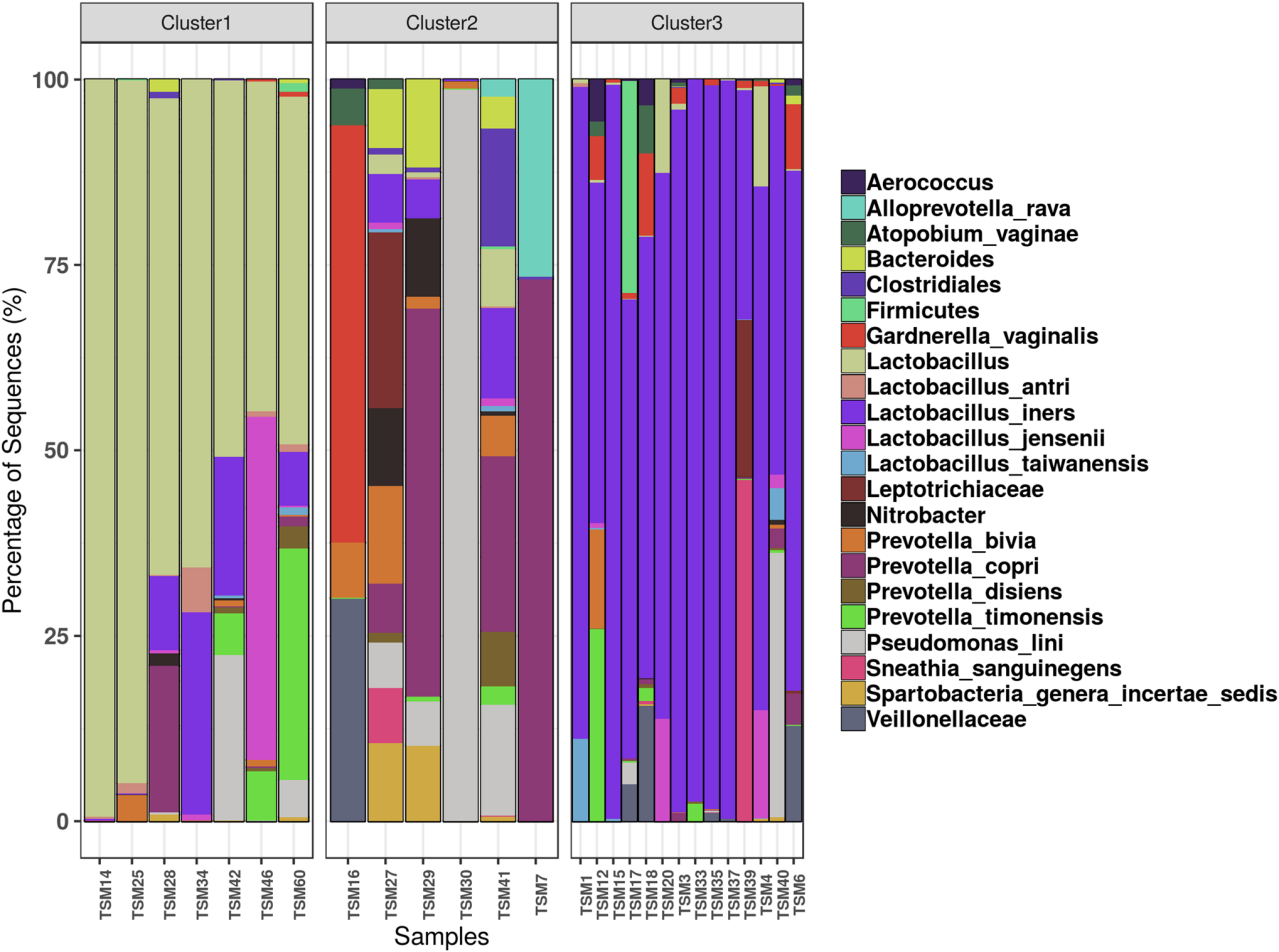
Bar plot presenting the relative abundance of the 30 most abundant OTUs of vaginal samples. OTUs were summarized at the highest taxonomy level with at least 80% confidence, into 22 different taxa. Each stacked bar represents the relative abundance of each vaginal maternal sample, grouped according to its respective community cluster

Vaginal microbial composition differed greatly between groups. On average mothers assigned to Cluster 1 had dominance of an unidentified species of *Lactobacillus* making up an average of 68.1% of the vaginal microbial community. Also, *L. iners*, *L. antri*, *L. jensenii*, *Prevotella timonensis*, *P. bivia* and *P. copri* were also detected (Fig. 3). This vaginal community could not be matched to a specific Community State Type (CST) described by Romero et al. (2014a, b). Women grouped into Cluster 2 was marked mainly by very low abundance, or absence, of *Lactobacillus* spp. and presence of *P. bivia*, *P. copri*, *P. disiens*, *Gardnerella vaginalis* and *Bacteroides* (Fig. 3). This vaginal community matches the CSTIV described by Romero et al. (2014a, b), which has low abundance of *Lactobacillus* spp. and high frequency and abundance of taxa related to bacterial vaginosis, such as *Gardnerella*, *Prevotella*, and *Atopobium*.

Moreover, more than half the women sampled were grouped into Cluster 3, which had the highest abundance of *L. iners* among the three vaginal community types, with mean abundance of 74.73%. And in addition to dominance of *L. iners*, *G. vaginalis*, *Atopobium vaginae, Sneathia sanguinegens* and *Veillonellaceae* were also found (Fig. 3). This description matches the CSTIII described by Romero et al. (2014a, b). Also, *L. iners* was present in all vaginal communities, though, with different overall mean abundance. Following the characterization of vaginal communities’ microbial profiles, we sought to find whether the different microbial composition also reflected on overall differences on hither phenotype characteristics. The BugBase (Ward et al. 2017) platform was applied for phenotype prediction. We found that differences on vaginal microbial composition also reflected on overall community phenotype characteristics. Cluster 1 had higher mean abundance of aerobic bacteria than Cluster 3, 50.19% and 4.99%, respectively (Table 3). Mean abundance of facultative anaerobic was different between all clusters, ranging from 5.78%, for Cluster 2, to 70%, for Cluster 3. There was no significant difference in abundance of anaerobic bacteria between vaginal communities. Also, Cluster 1 and 3 were mainly composed of gram-positive bacteria, 80.56 and 85.38% respectively, while Cluster 2 was composed of mainly gram-negative, 69.18% (Table 3).

**Table 3:** Predicted phenotypes of different vaginal microbial cluster found in this study

	Mean abundance (%)			p-Value		
	Cluster 1	Cluster 2	Cluster 3	Cluster 1 vs. Cluster 2	Cluster 1 vs. Cluster 3	Cluster 2 vs. Cluster 3
Aerobic	50.19	34.20	4.99	0.2948	0.00003*	0.2391
Anaerobic	13.35	53.09	24.21	0.1014	0.8557	0.0757
Facultative anaerobic	28.69	5.78	70.13	0.0011*	0.0022*	0.00005*
Gram-Negative	19.44	69.18	14.62	0.0081*	0.5352	0.0006*
Gram-Positive	80.56	30.82	85.38	0.0081*	0.5352	0.0006*
Mobile elements	11.28	19.15	2.87	0.945	0.0007*	0.1093

*Pairwise Mann–Whitney–Wilcoxon Test

### Newborn’s gut similarity with mother’s vaginal microbial community cluster

After finding and characterizing three different vaginal microbial community clusters in Brazilian mothers, we explored how much of those differences were associated with the composition of babies’ gut microbiota at birth.

**Fig. 4:**
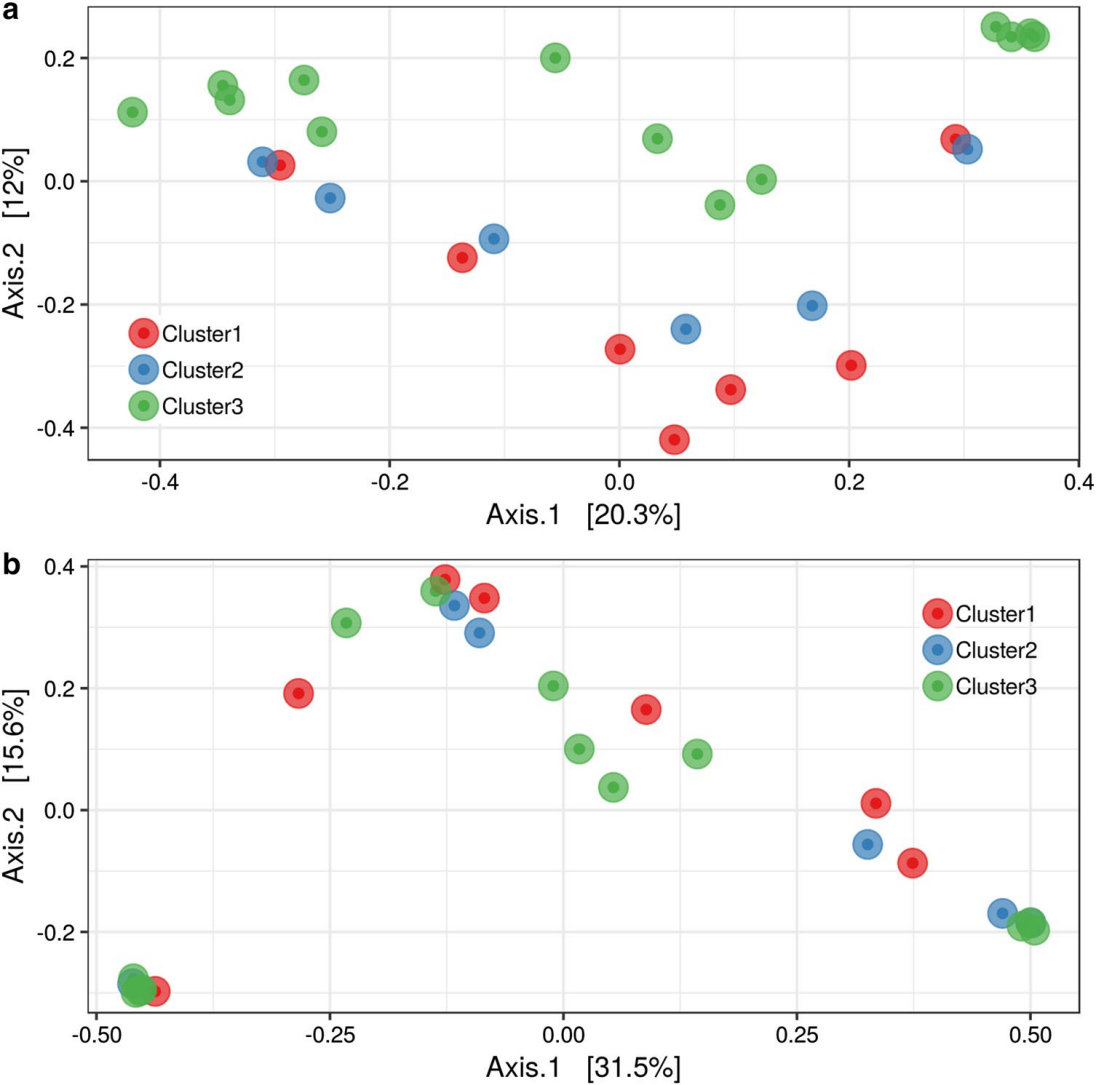
Beta diversity ordinated with MDS of microbial communities present in meconium samples. Each symbol represents a microbial community of an individual sample and each color represents a cluster assigned to newborns’ respective mother. **a** Binary distance of microbial communities, based on presence and absence. **b** Bray–Curtis dissimilarity

Beta diversity of infants’ samples was measured with Bray–Curtis dissimilarity and Binary distance, ordinated with MDS (Fig. 4), and tested with PERMANOVA. Overall, the three vaginal maternal clusters were sufficient to cluster infants’ samples regarding the presence and/or absence (p-value = 0.002), however explained little of the variation, with R^2^ of 15.8% (Fig. 4a). Bray–Curtis dissimilarity showed no difference, p-value 0.509, between infants from mothers from different clusters (Fig. 4b).

**Fig. 5:**
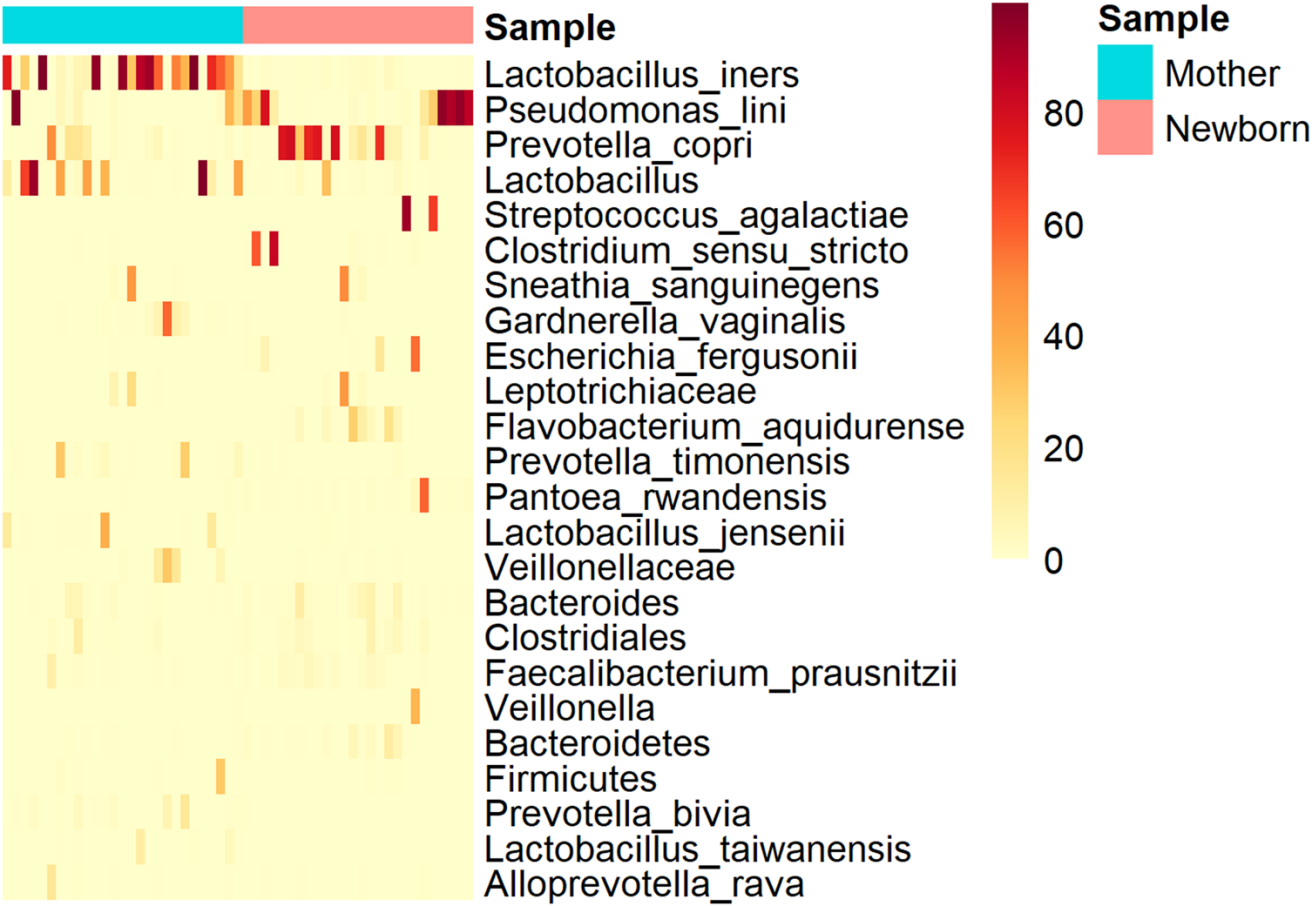
Heatmap with the most abundant taxa across maternal and infant microbiota. Each line represents a taxon, summarized at the highest taxonomy level, and each column represents an individual sample. These are the taxa with more than 10% relative abundance in at least one sample

In order to better examine how maternal vaginal microbial composition compared to the newborn’s gut composition, we constructed a heatmap with the most abundant taxa across mothers and infants. We found that there was not a clear clustering of these samples, with some common low frequent taxa in low abundance shared between them (Fig. 5), such as *Bacteroides*, *Clsotridiales* and *Faecalibacterium prausnitzii*. *Pseudomonas lini* and *Prevotella copri* were the shared taxa with the highest abundance in the newborns’ gut, and that were also present in the maternal vaginal bacterial community.

Considering the correlation of mothers’ microbes with the composition of the babies’ gut, we also found that common OTUs between mothers and infants differed between the clusters. Overall, pairs of babies and mothers form Cluster 1 shared 15 different OTUs, while Cluster 2 and 3 shared 25 and 7, respectively. Babies from Cluster 2 had 60.31% of their gut bacterial composition similar with the mother’s vaginal microbiota, while babies from the *Lactobacillus* dominated clusters, Cluster 1 and 3, had 32.54 and 50.32%, respectively (Table 4). Babies from Cluster 2 had the highest proportion of *Lactobacillus*, 5.67%, while babies from mothers on Cluster 1 and 3 had only 2.03 and 0.75%. In addition, on average babies from all three clusters had the same two OTUs, the same 200 nt sequence, as the most abundant of the shared OTUs and identified as *Pseudomonas lini* and *Prevotella copri*. The 30 most abundant OTUs comprising the gut microbiota of each newborn is presented in Supplementary Fig. S1.

**Table 4:** Relative mean abundance of shared OTUs between vaginal and meconium samples within each cluster

OTU ID	Taxonomy	Meconium (%)	Vaginal (%)
Cluster 1	15 Shared phylotypes		
OTU1	*Pseudomonas lini*	10.97	3.46
OTU2	*Prevotella copri*	8.10	1.01
OTU3	*Prevotella copri*	3.53	0.46
OTU4	Bacteroides	3.08	0.11
OTU5	*Prevotella copri*	2.23	0.21
OTU6	Lactobacillus	1.09	55.78
OTU7	Nitrobacter	1.02	0.25
OTU8	*Lactobacillus iners*	0.77	8.07
OTU9	Alistipes	0.45	0.06
OTU10	Lachnospiraceae	0.32	0.22
OTU11	*Prevotella timonensis*	0.28	5.52
OTU12	*Spartobacteria genera incertae sedis*	0.21	0.08
OTU13	Firmicutes	0.21	0.21
OTU14	Lactobacillus	0.17	1.65
OTU15	Flavobacterium	0.11	0.18

Cluster 2	25 Shared phylotypes		
OTU1	*Pseudomonas lini*	32.53	18.02
OTU2	*Prevotella copri*	7.73	4.22
OTU6	Lactobacillus	4.66	0.44
OTU5	*Prevotella copri*	3.06	1.24
OTU3	*Prevotella copri*	2.43	6.73
OTU4	Bacteroides	1.68	1.37
OTU16	*Bacteroides uniformis*	1.31	0.44
OTU8	*Lactobacillus iners*	1.01	1.36
OTU17	*Faecalibacterium prausnitzii*	0.90	0.88
OTU18	Clostridiales	0.88	1.42
OTU19	*Parabacteroides merdae*	0.69	0.16
OTU20	*Bacillus bataviensis*	0.41	0.25
OTU21	*Prevotella copri*	0.34	0.23
OTU9	Alistipes	0.34	0.33
OTU22	Bacillus	0.31	0.23
OTU23	*Spartobacteria genera incertae sedis*	0.29	0.98
OTU32	Ruminococcaceae	0.28	0.10
OTU24	*Faecalibacterium prausnitzii*	0.26	0.44
OTU25	*Prevotella copri*	0.26	0.16
OTU26	Leptotrichiaceae	0.23	0.93
OTU27	Gp1	0.20	0.36
OTU11	*Prevotella timonensis*	0.18	0.41
OTU28	Pseudomonas	0.16	0.57
OTU29	*Faecalibacterium prausnitzii*	0.11	0.72
OTU30	*Bacteroides coprocola*	0.05	0.33

Cluster 3	7 Shared phylotypes		
OTU1	*Pseudomonas lini*	22.50	2.68
OTU2	*Prevotella copri*	14.93	0.19
OTU3	*Prevotella copri*	4.43	0.46
OTU31	*Sneathia sanguinegens*	4.00	3.24
OTU26	Leptotrichiaceae	3.70	1.54
OTU8	*Lactobacillus iners*	0.57	69.82
OTU6	Lactobacillus	0.18	0.67

Caution is warranted regarding detection of *Pseudomonas lini*, as it’s commonly found on soil samples, and should be considered part of the “kitome” (Salter et al. 2014).

## Discussion

The Brazilian population is composed mainly by European, African and Amerindian ancestry. However, race in Brazil does not refer directly to ancestry rather it refers mostly to phenotype, such as skin color. This ambiguity nature of race in Brazil allows for individuals drift from one racial category to another, for example siblings and parents can often identify themselves as member of different racial groups (Telles 2004). This ambiguity is due to the highly miscegenation of the Brazilian population and therefore skin color becomes irrespective of ancestry. Recent research have shown that European ancestry in the Brazilian population is larger than expected, ranging between 60 to 77% depending on the region (Pena et al. 2011; Rodrigues de Moura et al. 2015). However, when Parra et al. (2003) compared white Brazilians with Portuguese (Europeans) and black Brazilians with Africans, they found that these populations were statistically different regarding to the alleles surveyed. They also found extensive overlaps in the African ancestry index among white, intermediate (pardos/brown) and blacks. Therefore, it is imprudent to use the standard stratifications of Caucasian/white and black/African Americans with the Brazilian population. It also strengthens the need to independently investigate populations with high miscegenation rates.

The vaginal microbiota changes rapidly over time, and fluctuations may occur in a matter of days (Gajer et al. 2012). Here we described the vaginal microbiota of pregnant healthy Brazilian mothers, right before delivery. To our knowledge, there is few descriptions of the vaginal microbiota of laboring mothers, immediately before delivery (Martín et al. 2007; Avershina et al. 2017).

A literature review of studies addressing the vaginal microbial communities at third trimester of pregnancy is presented in Table 5. *Lactobacillus* spp. were detected in higher frequency and in higher abundance among all women irrespective of the women background. However, a low proportion of pregnant women presented a vaginal microbial community that was not dominated by *Lactobacillus*. Those women did not present clinical symptoms of vaginosis, their vaginal microbial community was more diverse (greater number of taxa) and presented greater abundance of *Atopobium*, *Pseudomonas*, *Gardnerella* and *Prevotella*.

**Table 5:** Review of studies addressing the vaginal microbial communities at the third trimester of pregnancy

Authors	Country	Type of study and methodology	Study characteristics	Most abundant taxa
Avershina et al. (2017)	Norway	Randomized double blind clinical trial V3–V4 hypervariable region of the 16 S rRNA	256 pregnant women with term gestation	*L. iners, L. crispatus, Enterobacteriaceae and Prevotella*
Chu et al. (2017)	USA	Prospective cohort study V5–V3 hypervariable region of the 16S rRNA gene	81 pregnant women with term gestation	*Lactobacillus* spp., * Prevotella* spp., *Streptococcus* spp., *Corynebacterium* spp.
MacIntyre et al. (2015)	UK	Longitudinal study V1–V2 hypervariable regions of 16S rRNA gene	42 pregnant women with term gestation; 23 (54.8%) White, 5 (11.9%) Black; 13 (31%) Asian	*Lactobacillus crispatus, L. iners, L. jensenii, L. gasseri, Prevotella* spp.
Bisanz et al. (2015)	Tanzania	Longitudinal open-label study V4 hypervariable region of 16S rRNA gene	56 pregnant women, 53 with term gestation	*Lactobacillus* spp., *Prevotella* spp., *Gardnerella* spp., *Sneathia* spp.
Romero et al. (2014a)	USA	Retrospective case–control longitudinal study V1–V2 hypervariable regions of 16S rRNA gene	22 pregnant women with term gestation; 19 (86%) African American, 2 (9%) White, 1 (5%) Hispanic	*L. iners, L. crispatus, Atopobium vaginae, Lactobacillus, L. Jensenii*
Romero et al. (2014b)	USA	Nested case–control study V1-V3 hypervariable regions of 16S rRNA gene	72 pregnant women with term gestation; 62 (86.1%) African American, 4 (5.6%) White, 6 (8.3%) Others	*L. iners, L. crispatus, Gardnerella vaginalis, L. Jensenii,* BVAB1
Hernández-Rodríguez et al. (2011)	Mexico	Transversal study V3 hypervariable region of 16S rRNA gene	23 pregnant women with term gestation	*L. acidophilus, L. iners, Ureaplasma urealyticum, L. gasseri,* BVAB1
This work	Brazil	Cohort study V4 hypervariable region of 16S rRNA gene	27 pregnant women with term gestation	*L. iners, Lactobacillus, Pseudomonas lini, G. vaginalis, Prevotella copri*

In this study, we found three different vaginal microbial community assemblies in Brazilian mothers at their third trimester of a healthy gestation. The Cluster 3 found here, matches the descriptions of the CSTIII (Community State Type) described in other populations, which has dominance of *Lactobacillus iners*. Cluster 2 matches descriptions of the CSTIV, which has low abundance of *Lactobacillus* spp. and high frequency and abundance of taxa related to bacterial vaginosis, such as *Gardnerella*, *Prevotella*, and *Atopobium* (Romero et al. 2014a, b). However, our Cluster 1, dominated by unidentified species of *Lactobacillus* spp., can be matched to any other CSTs dominated by *Lactobacillus* spp. (*L*. *crispatus*, *L*. *gaseri*, *L*. *jensenii*) described in the literature, even though it presented low abundance of *L*. *antri*, *L. iners*, *L*. *jensenii*. Nevertheless, despite the similarities of the community clusters dominated by *Lactobacillus* with the others already described, the clusters dominated by *Lactobacillus* found in Brazilian mothers had prevalence, albeit low abundance, of bacterial vaginosis associated bacteria. The majority of the sampled Brazilian mothers presented a cluster dominated by *Lactobacillus spp.* About half (51.8%) of the mothers had dominance of *L. iners*, which are in consonance with both reports (59.4% and 59.1%) from USA (Romero et al. 2014a, b), though reports from UK had lower rates (31%) of dominance of *L. iners*. It is important to highlight the high proportions of black women in both reports from USA (90% and 86%). The prevalence of the diverse cluster in Brazilian mothers was the same as one of the reports form USA, 22%, however it were much higher compared to UK and another report from USA, 2.4% and 0%, respectively (Romero et al. 2014b; MacIntyre et al. 2015). Although our primers are able to amplify *L. crispatus* and *L. gasseri*, these two microbial species were not detected in our samples. They might not be present in our dataset or their abundance was below the detection level of our technique.

During pregnancy, increasing levels of oestrogen lead to the maturation of the vaginal epithelium and accumulation of glycogen, which is broken down into maltose, maltotriose, and maltotetraose supporting *Lactobacillus* spp. colonization (Spear et al. 2014). This increase in oestrogen levels is thought to drive the increase in proportion of *Lactobacillus* spp. in the vagina throughout pregnancy. On the other hand, Avershina et al. (2017) investigated the vaginal microbiota of women at labor and found that by the time of labor onset the number of observed species are increased. In particular, the phylotypes that are characteristic of CST IV (*Peptoniphilus, Anaerococcus, Corynebacterium, Finegoldia, Prevotella*) were overrepresented at labor. This supports our findings, that even the vaginal microbial communities dominated by *Lactobacillus* spp. had considerable abundance of BV related bacteria at labor onset.

The uterine environment has been considered sterile, in which babies were thought to be born sterile, acquiring their gut microbial community after birth. Recent several studies have described the microbial composition of first pass meconium (Jiménez et al. 2008; Mshvildadze et al. 2010; Madan et al. 2012; Dobbler et al. 2017), placenta (Aagaard et al. 2014) and amniotic fluid (Collado et al. 2016) suggesting that microbial seeding of the fetus gut might occur before birth. Overall, the mothers’ vaginal microbial community cluster at time of labor was associated with the microbial presence and absence in the gut microbiota of their newborn at birth, though not strong enough to affect the community structure. Reflecting the different composition of each vaginal community type, common OTUs between mothers and babies were also different.

More than half of the composition of the babies’ gut microbiota of mothers from Cluster 2 was found in their mothers, which could be a result of the higher diversity. Even though *Lactobacillus* spp. were most frequently the most abundant in the vagina, it was in very low abundance in the meconium samples, while OTUs identified as *Pseudomonas lini* and *Prevotella copri* were the most frequently shared and abundant in the babies’ gut at time of birth. Low resemblance of the newborn gut with the maternal vaginal microbiota have been recently reported in vaginally delivered babies. It was suggested that babies receive microbes from several maternal body sites, though the microbes from maternal gut were more persistent (Ferretti et al. 2018). The OTUs shared between babies and mothers of different clusters, might reflect on initial colonizers of the developing newborns’ gut.

The reads obtained by high throughput 16S rRNA gene sequencing surveys represent a random sample of the relative abundance of DNA molecules. According to Gloor et al. (2017), due to the nature of the data it cannot be related to the absolute number of microbes in a sample. The data presenting such random component are referred to as compositional (Aitchison et al. 2000; Gloor and Reid 2016) and the multivariate approaches, usually applied in microbial ecology studies, such as ordination and clustering are considered inappropriate (Pawlowsky-Glahn et al. 2015). While the arguments in favor of compositional analyses are plausible, most tools available for microbiome analysis do not take into account the compositionality of the data. This opens a discussion on whether or not any other work based on non-compositional models should be rejected. Here we reanalyzed our results using a compositional approach described by Gloor and Reid (2016) by converting 16S rRNA counts using the centered log-ratio (clr) transformation. The results are presented in the supplementary material (Supplementary Data S1) of this manuscript. For our particular dataset the same biological conclusion was reached irrespective of the approach chosen for data analysis.

## Conclusion

Here, we characterize three different vaginal microbial community types found in Brazilian mothers at time of labor. Two of these community types were dominated by *Lactobacillus* spp. and one was marked by lower abundance of *Lactobacillus* spp. and higher abundance of BV related bacteria. Irrespective of cluster membership, vaginosis related bacteria were frequently found in Brazilian mothers. Other community types were not detected in this cohort and might be due to our small number of women sampled here. In addition, the vaginal microbiota showed significant association with presence of microbes in the babies’ gut at the time of birth. On the other hand, high abundance of those vaginal microbes did not correlate with high abundance in the infant’s gut microbiota. Overall, maternal vaginal microbiota had low resemblance with initial baby’s gut colonization, and maternal vaginal clusters dominated with *Lactobacillus* were not associated with *Lactobacillus* in the babies’ meconium at time of birth.

## Electronic supplementary material

Below is the link to the electronic supplementary material.

Supplementary material 1 Supplementary Fig. 1. Bar plot presenting the relative abundance of the 30 most abundant OTUs across all meconium samples. OTUs were summarized at the highest taxonomy level with at least 80% confidence, into 24 different taxa. Each stacked bar represents the relative abundance of each subject grouped according to respective mother’s community cluster (TIF 539 kb)

Supplementary material 2 (DOCX 15 kb)

Supplementary material 3 (DOCX 14 kb)

Supplementary material 4 (DOCX 15 kb)

Supplementary material 5 (DOCX 15 kb)

Supplementary material 6 (PDF 349 kb)
