# Supplementary figures and images for: The vaginal microbial communities of healthy expectant Brazilian mothers and its correlation with the newborn’s gut colonization
Source: World J Microbiol Biotechnol. 2019 Oct 10;35(10):159. doi: 10.1007/s11274-019-2737-3 (PMC6787113; doi:10.1007/s11274-019-2737-3)

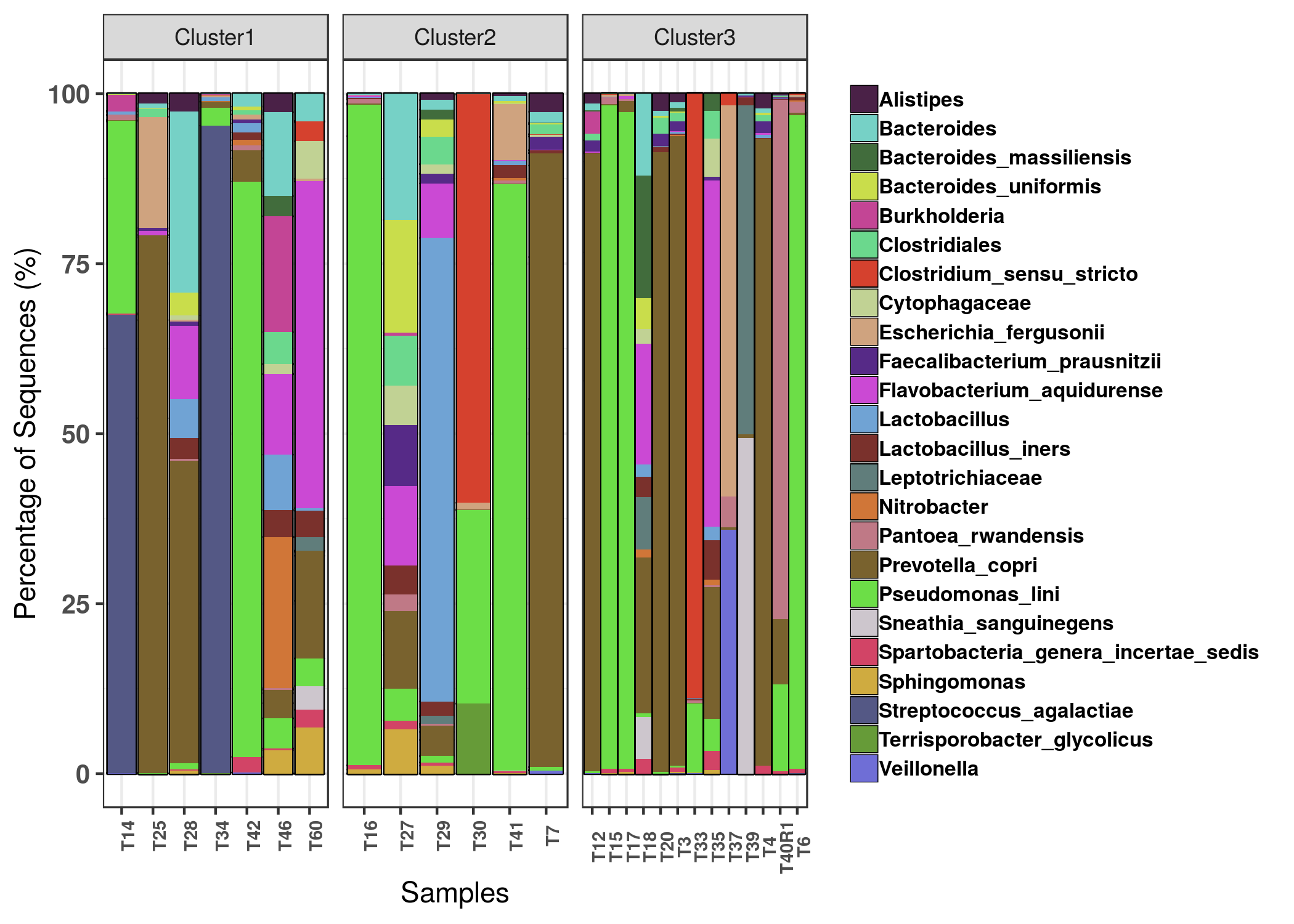

Supplement: Supplementary file 1 — Supplementary material 1 Supplementary Fig. 1. Bar plot presenting the relative abundance of the 30 most abundant OTUs across all meconium samples. OTUs were summarized at the highest taxonomy level with at least 80% confidence, into 24 different taxa. Each stacked bar represents the relative abundance of each subject grouped according to respective mother’s community cluster (TIF 539 kb) [file 11274_2019_2737_MOESM1_ESM.tif]
