# Supplementary material for: The vaginal microbial communities of healthy expectant Brazilian mothers and its correlation with the newborn’s gut colonization
Source: World J Microbiol Biotechnol. 2019 Oct 10;35(10):159. doi: 10.1007/s11274-019-2737-3 (PMC6787113; doi:10.1007/s11274-019-2737-3)
Supplement: Supplementary file 2 — Supplementary material 2 (DOCX 15 kb) [file 11274_2019_2737_MOESM2_ESM.docx]

**Table S1:**

**Good´s Coverage of vaginal and meconium samples.**

| Sample ID | Good´s Coverage |
| --- | --- |
| T12 | 0.993048 |
| T14 | 0.990228 |
| T15 | 0.986094 |
| T16 | 0.986679 |
| T17 | 0.988706 |
| T18 | 0.997921 |
| T20 | 0.995617 |
| T25 | 0.991899 |
| T27 | 0.995997 |
| T28 | 0.991366 |
| T29 | 0.997876 |
| T3 | 0.993286 |
| T30 | 0.9936 |
| T33 | 0.994281 |
| T34 | 0.99677 |
| T35 | 0.996362 |
| T37 | 0.997765 |
| T39 | 0.997075 |
| T4 | 0.98986 |
| T40R1 | 0.965497 |
| T41 | 0.986689 |
| T42 | 0.991156 |
| T46 | 0.991174 |
| T6 | 0.981714 |
| T60 | 0.996223 |
| T7 | 0.995467 |
| TSM1 | 0.999359 |
| TSM12 | 0.99973 |
| TSM14 | 0.994602 |
| TSM15 | 0.999439 |
| TSM16 | 0.998652 |
| TSM17 | 0.992515 |
| TSM18 | 0.999856 |
| TSM20 | 0.999559 |
| TSM25 | 0.991758 |
| TSM27 | 0.983056 |
| TSM28 | 0.946063 |
| TSM29 | 0.89324 |
| TSM3 | 0.991801 |
| TSM30 | 0.995738 |
| TSM33 | 0.999089 |
| TSM34 | 0.999601 |
| TSM35 | 0.998981 |
| TSM37 | 0.999438 |
| TSM39 | 0.99607 |
| TSM4 | 0.990185 |
| TSM40 | 0.971064 |
| TSM41 | 0.912029 |
| TSM42 | 0.947406 |
| TSM46 | 0.998593 |
| TSM6 | 0.996111 |
| TSM60 | 0.951807 |
| TSM7 | 0.977384 |
