# Supplementary material for: The vaginal microbial communities of healthy expectant Brazilian mothers and its correlation with the newborn’s gut colonization
Source: World J Microbiol Biotechnol. 2019 Oct 10;35(10):159. doi: 10.1007/s11274-019-2737-3 (PMC6787113; doi:10.1007/s11274-019-2737-3)
Supplement: Supplementary file 3 — Supplementary material 3 (DOCX 14 kb) [file 11274_2019_2737_MOESM3_ESM.docx]

| Hopkins Statistic Test | | | | | | | |
| --- | --- | --- | --- | --- | --- | --- | --- |
| H value | | | | 0.19 | | | |
| Gap Statistics Analysis | | | | | | | |
| Number of clusters | Log of within cluster sum of squares | | e. Log of within cluster sum of squares | | Gap statistic value (e.logW-logW) | | Standard error |
| 1 | 1.615 | | 1.824 | | 0.209 | | 0.031 |
| 2 | 1.380 | | 1.718 | | 0.338 | | 0.035 |
| 3 | 1.217 | | 1.624 | | 0.407 | | 0.037 |
| 4 | 1.154 | | 1.538 | | 0.383 | | 0.037 |
| 5 | 1.049 | | 1.455 | | 0.406 | | 0.039 |
| 6 | 0.965 | | 1.375 | | 0.410 | | 0.040 |
| 7 | 0.842 | | 1.296 | | 0.454 | | 0.042 |
| 8 | 0.750 | | 1.220 | | 0.470 | | 0.043 |
| K-means Analysis | | | | | | | |
|  | | Cluster 1 | | Cluster 2 | | Cluster 3 | |
| Within cluster sum of squares by cluster | | 1.379 | | 0.926 | | 2.050 | |
|  | |  | |  | |  | |

**Table S2**:

**Unsupervised clustering analysis of vaginal microbiota of Brazilian mothers.**
