# Supplementary material for: The vaginal microbial communities of healthy expectant Brazilian mothers and its correlation with the newborn’s gut colonization
Source: World J Microbiol Biotechnol. 2019 Oct 10;35(10):159. doi: 10.1007/s11274-019-2737-3 (PMC6787113; doi:10.1007/s11274-019-2737-3)
Supplement: Supplementary file 4 — Supplementary material 4 (DOCX 15 kb) [file 11274_2019_2737_MOESM4_ESM.docx]

| **Analysis of Variance** | | | | |
| --- | --- | --- | --- | --- |
|  | Df | Mean Squares | F value | P-value |
| Groups (clusters) | 2 | 0.203113 | 8.6186 | 0.00151 |
| Residuals | 24 | 0.023567 |  |  |
|  |  |  |  |  |
| **Post-hoc (Tukey’s Honest Significant Differences)** | | | | |
|  | Difference | Lower | Upper | P-value adj. |
| Cluster2-Cluster1 | 0.234658 | 0.02137 | 0.447946 | 0.029117 |
| Cluster3-Cluster1 | -0.07574 | -0.25321 | 0.101725 | 0.543866 |
| Cluster3-Cluster2 | -0.3104 | -0.49747 | -0.12333 | 0.00103 |

**Table S3:**

**Multivariate homogeneity of groups dispersions.**

Lower and upper end point of the 95% Confidence Interval.
