# Supplementary material for: The vaginal microbial communities of healthy expectant Brazilian mothers and its correlation with the newborn’s gut colonization
Source: World J Microbiol Biotechnol. 2019 Oct 10;35(10):159. doi: 10.1007/s11274-019-2737-3 (PMC6787113; doi:10.1007/s11274-019-2737-3)
Supplement: Supplementary file 5 — Supplementary material 5 (DOCX 15 kb) [file 11274_2019_2737_MOESM5_ESM.docx]

|  |  | **Mean Abundance (%)** | | | **p-value**** | | | | |
| --- | --- | --- | --- | --- | --- | --- | --- | --- | --- |
| **KEGG_Pathways** | **Effect size*** | **Cluster 1** | **Cluster 2** | **Cluster 3** | | **Cluster 1 vs Cluster 2** | | **Cluster 1 vs Cluster 3** | **Cluster 2 vs Cluster 3** |
| Excretory System | 0.602 | 0.03 | 0.03 | 0.00 | ≥0.1 | | <0.001 | | <0.01 |
| Metabolism of Terpenoids and Polyketides | 0.578 | 1.82 | 1.99 | 1.59 | ≥0.1 | | <0.01 | | <0.001 |
| Transport and Catabolism | 0.573 | 0.17 | 0.31 | 0.12 | <0.01 | | ≥0.1 | | <0.001 |
| Endocrine System | 0.561 | 0.19 | 0.36 | 0.18 | <0.001 | | ≥0.1 | | <0.001 |
|  |  |  |  |  |  | |  | |  |
| Amino Acid Metabolism | 0.550 | 7.61 | 9.94 | 7.02 | <0.01 | | ≥0.1 | | <0.001 |
| Immune System Diseases | 0.507 | 0.09 | 0.06 | 0.10 | <0.01 | | ≥0.1 | | <0.001 |
| Nervous System | 0.502 | 0.10 | 0.12 | 0.08 | ≥0.1 | | <0.02 | | <0.001 |
| Membrane Transport | 0.486 | 14.41 | 11.32 | 14.84 | <0.01 | | ≥0.1 | | <0.001 |
| Metabolism of Cofactors and Vitamins | 0.454 | 3.29 | 4.27 | 3.21 | <0.01 | | ≥0.1 | | <0.001 |
| Biosynthesis of Other Secondary Metabolites | 0.432 | 0.65 | 0.92 | 0.54 | <0.05 | | ≥0.1 | | <0.001 |
| Metabolism | 0.358 | 2.47 | 2.49 | 2.11 | ≥0.1 | | <0.02 | | <0.02 |
| Carbohydrate Metabolism | 0.352 | 10.50 | 9.46 | 10.46 | <0.02 | | ≥0.1 | | <0.01 |

**Table S4:**

**Relative abundance of predicted microbial genes using PICRUSt.**

*Only predictions with at least 0.35 of effect size
**Tested using Kruskal-Wallis followed by Tukey-Kramer *post hoc* test
