# Supplementary material for: The vaginal microbial communities of healthy expectant Brazilian mothers and its correlation with the newborn’s gut colonization
Source: World J Microbiol Biotechnol. 2019 Oct 10;35(10):159. doi: 10.1007/s11274-019-2737-3 (PMC6787113; doi:10.1007/s11274-019-2737-3)
Supplement: Supplementary file 6 — Supplementary material 6 (PDF 349 kb) [file 11274_2019_2737_MOESM6_ESM.pdf]

Compositional data analysis of the article: The vaginal microbial communities of healthy expectant Brazilian mothers and its correlation with the newborn's gut colonization

Article title: The vaginal microbial communities of healthy expectant Brazilian mothers and its correlation with the newborn's gut colonization

Authors: Priscila Dobbler, Volker Mai, Renato S Procianoy, Rita C Silveira, Andréa L Corso, Luiz Fernando Wurdig Roesch

Correspoonding author: Luiz Fernando Wurdig Roesch Centro Interdisciplinar de Pesquisas em Biotecnologia – CIP-Biotec, Campus São Gabriel, Universidade Federal do Pampa, São Gabriel, Rio Grande do Sul, Brazil luizroesch@unipampa.edu.br

Finding maternal vaginal community types

Only OTUs with at least 5 sequence reads were kept, and rarefied to 1020 sequences per sample.

```
library(microbiome)
library(phyloseq)
library(tidyverse)
jsonbiomfileM = "/Users/prisc/Desktop/Desktop.asus/seq21_RDP/teste/final/otu_tab_tax.biom"
mapfileM = "/Users/prisc/Desktop/Desktop.asus/paper_mothers_newborns/mothers/rdp/r/map.txt"
biomM = import_biom(jsonbiomfileM, mapfileM, parseFunction= parse_taxonomy_greengenes)
mapM = import_qiime_sample_data(mapfileM)
inputM = merge_phyloseq(biomM, mapM)
inputM = prune_taxa(taxa_sums(inputM) > 5, inputM)
set.seed(2125)
inputM=subset_taxa(inputM,Phylum!="" & Phylum!="Cyanobacteria/Chloroplast")
inputRM = rarefy_even_depth(inputM, sample.size = 1020)
tax_table(inputRM)=tax_table(inputRM)[,-8]
x=as.data.frame(tax_table(inputRM))
LastRank = function(x, ans = rep_len(NA, length(x[[1L]])), wh = seq_len(length(x[[1L]])))
{
  if(!length(wh)) return(ans)
  ans[wh] = as.character(x[[length(x)]])[wh]
  Recall(x[-length(x)], ans, wh[is.na(ans[wh])])
}
p=as.character(LastRank(as.list(x))) %>% make.unique(.)
tax_table(inputRM)=cbind(tax_table(inputRM), LastRank=p)
theme_set(theme_bw())
inputRM
```

```
## phyloseq-class experiment-level object
## otu_table()   OTU Table:         [ 683 taxa and 27 samples ]
## sample_data() Sample Data:       [ 27 samples by 6 sample variables ]
## tax_table()   Taxonomy Table:    [ 683 taxa by 8 taxonomic ranks ]
```

Centered log ratio transformation and cluster tendency

Data was transformed in centered log ratios (clr) using the microbiome package, function 'transform'. Euclidean distance was computed and used for cluster tendency assessment, number and membership of clusters. Cluster tendency was accessed with visual inspection and Hopkins statistics, where values closest to zero there are cluster tendency.

```
#CLR transformation
library(microbiome)
inputm.clr=transform(inputRM, "clr")
d3 = as.matrix(distance(inputm.clr, "euclidean"))
factoextra::get_clust_tendency(d3, n=26, graph = F)
```

```
## $hopkins_stat
## [1] 0.2971269
##
## $plot
## NULL
```

Number clusters and samples assignment

The distance of Aitchison (Euclidean distance applied to CLR transformed data) was used for clustering analysis. Gap statistic was performed with 500 Monte Carlo simulations. The members of each cluster were then identified using k-means with the number of clusters derived from the previous analysis, with 25 different random starting assignments.

```
library(cluster)
pam1 <- function(x,k) list(cluster = pam(x,k, cluster.only=TRUE))
gskmn = clusGap(d3, FUN=pam1, K.max = 6, B = 500)
gskmn
```

```
## Clustering Gap statistic ["clusGap"] from call:
## clusGap(x = d3, FUNcluster = pam1, K.max = 6, B = 500)
## B=500 simulated reference sets, k = 1..6; spaceH0="scaledPCA"
##  --> Number of clusters (method 'firstSEmax', SE.factor=1): 3
##          logW   E.logW       gap     SE.sim
## [1,] 5.542254 5.722157 0.1799034 0.03884793
## [2,] 5.267781 5.507056 0.2392748 0.02941028
## [3,] 5.136507 5.418577 0.2820693 0.02968304
## [4,] 5.060468 5.343590 0.2831220 0.02912477
## [5,] 4.972472 5.272683 0.3002109 0.02868342
## [6,] 4.897173 5.203047 0.3058742 0.02892239
```

```
####KMEANS CLUSTERING
km.res <- kmeans(d3, 3, nstart = 25)
clusters=km.res$cluster
clusters=sub("^","Cluster ",clusters)
sample_data(inputm.clr)=cbind(sample_data(inputm.clr), Clusters_kmeans=clusters)
library(kableExtra)
kable(table(clusters)) %>% kable_styling()
```

clusters	Freq
Cluster 1	14
Cluster 2	8
Cluster 3	5

Association between maternal vaginal microbiome and newborn's gut

Infants' meconium microbiota. Data was filtered, rarefied and transformed as the maternal samples.

```
jsonbiomfile = "/Users/prisc/Desktop/Desktop.asus/seq21_RDP/teste/final/otu_tab_tax.biom"
mapfileN = "/Users/prisc/Desktop/Desktop.asus/paper_mothers_newborns/mothersPlusNewborns/TermNewborns/ma
biomN = import_biom(jsonbiomfile, mapfileN, parseFunction= parse_taxonomy_greengenes)
mapN = import_qiime_sample_data(mapfileN)
inputN = merge_phyloseq(biomN, mapN)
inputN = prune_taxa(taxa_sums(inputN) > 5, inputN)
set.seed(2125)
inputN
```

```
## phyloseq-class experiment-level object
## otu_table()   OTU Table:         [ 975 taxa and 26 samples ]
## sample_data() Sample Data:       [ 26 samples by 12 sample variables ]
## tax_table()   Taxonomy Table:    [ 975 taxa by 8 taxonomic ranks ]
```

```
inputN=subset_taxa(inputN,Phylum!="" & Phylum!="Cyanobacteria/Chloroplast")
inputRN = rarefy_even_depth(inputN, sample.size = 1020)
tax_table(inputRN)=tax_table(inputRN)[,-8]
#############LastRank----
x=as.data.frame(tax_table(inputRN))
LastRank = function(x, ans = rep_len(NA, length(x[[1L]])), wh = seq_len(length(x[[1L]])))
{
  if(!length(wh)) return(ans)
  ans[wh] = as.character(x[[length(x)]])[wh]
  Recall(x[-length(x)], ans, wh[is.na(ans[wh])])
}
p=as.character(LastRank(as.list(x))) %>% make.unique(.)
tax_table(inputRN)=cbind(tax_table(inputRN), LastRank=p)
rn.clr=transform(inputRN, "clr")
rn.euclid=as.matrix(distance(rn.clr, "euclidean"))
```

Alpha diversity

Alpha diversity of maternal and infants' samples. For alpha diversity was computed on the non-transformed filtered data set, using the clusters found with the clr transformed data.

```
library(knitr)
tab <- global(inputM, index = c("Shannon", "Observed"))
##add diversity to metadata
ps1.meta <- meta(inputm.clr)
ps1.meta$Shannon <- tab$diversities_shannon
ps1.meta$Observed <- tab$observed
# create a list of pairwise comaprisons
clusters <- levels(ps1.meta$Cluster) # get the variables
# make a pairwise list that we want to compare.
clusters.pairs <- combn(seq_along(clusters), 2, simplify = FALSE,
                        FUN = function(i)clusters[i])
library(ggpubr)
p1 <- ggviolin(ps1.meta, x = "Cluster", y = "Shannon",
               add = "boxplot", fill = "Cluster",
```

```r
               palette = c("#a6cee3", "#b2df8a", "#fdbf6f"), xlab ="")
p1 <- p1 + stat_compare_means(comparisons = clusters.pairs) +
  stat_compare_means(label.y =9) + rremove("x.text")
p2 <- ggviolin(ps1.meta, x = "Cluster", y = "Observed",
               add = "boxplot", fill = "Cluster",
               palette = c("#a6cee3", "#b2df8a", "#fdbf6f"), xlab = "")
p2 <- p2 + stat_compare_means(comparisons = clusters.pairs) +
  stat_compare_means(label.y =650)+ rremove("x.text")
#infants
tab <- global(inputN, index = c("Shannon", "Observed"))
##add diversity to metadata
ps1.meta <- meta(rn.clr)
ps1.meta$Shannon <- tab$diversities_shannon
ps1.meta$Observed <- tab$observed
# create a list of pairwise comaprisons
clusters <- levels(ps1.meta$ClusterM) # get the variables
# make a pairwise list that we want to compare.
clusters.pairs <- combn(seq_along(clusters), 2, simplify = FALSE,
                        FUN = function(i)clusters[i])
p3 <- ggviolin(ps1.meta, x = "ClusterM", y = "Shannon",
               add = "boxplot", fill = "ClusterM",
               palette = c("#a6cee3", "#b2df8a", "#fdbf6f"), xlab ="")
p3 <- p3 + stat_compare_means(comparisons = clusters.pairs) +
  stat_compare_means(label.y =9) + rremove("x.text")
p4 <- ggviolin(ps1.meta, x = "ClusterM", y = "Observed",
               add = "boxplot", fill = "ClusterM",
               palette = c("#a6cee3", "#b2df8a", "#fdbf6f"), xlab = "")
p4 <- p4 + stat_compare_means(comparisons = clusters.pairs) +
  stat_compare_means(label.y =650)+ rremove("x.text")
ggarrange(p2,p1,p4,p3, labels = c("AUTO"), common.legend = T, legend = "bottom")
```

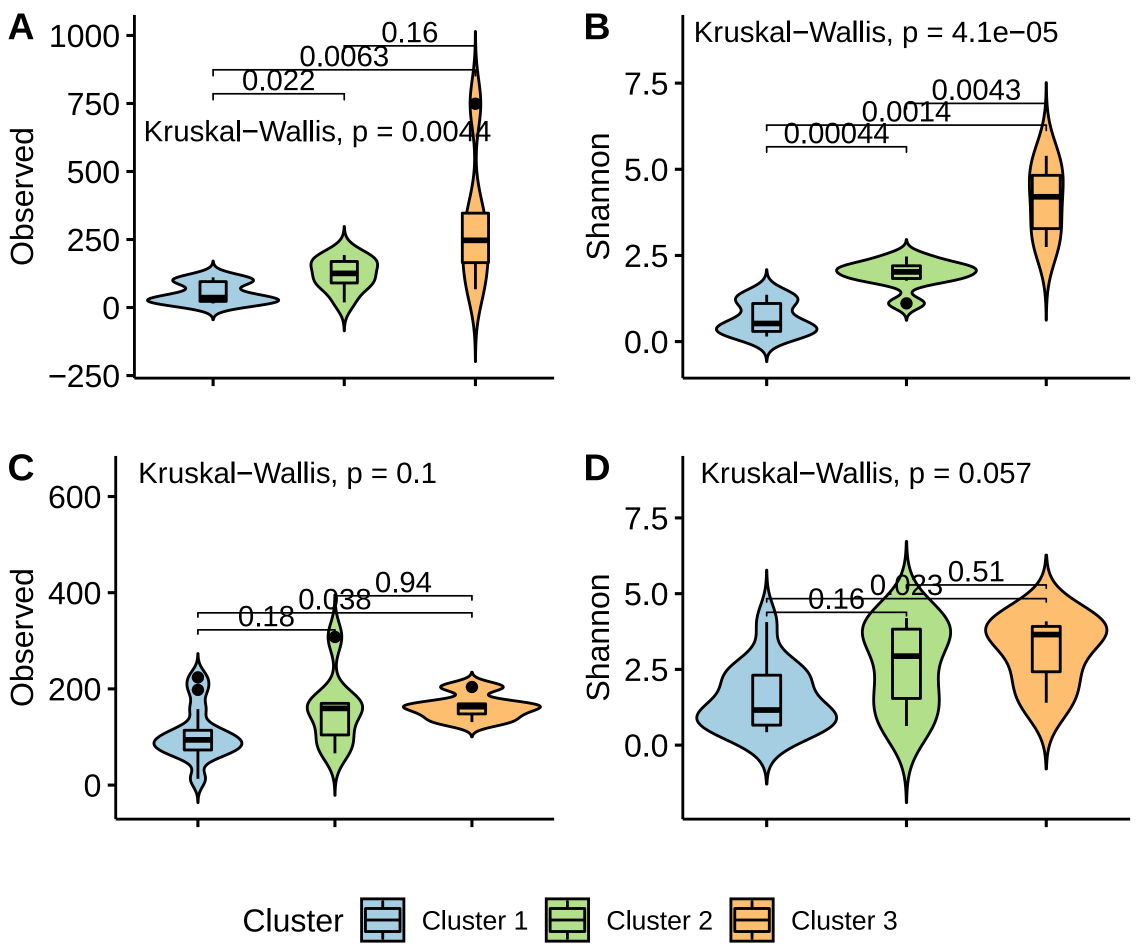

Figure 1: Alpha diversity of maternal and newborns' samples: A) and B) Alpha diversity of maternal vaginal microbiota; C) and D) Alpha diversity of newborns' gut microbiota at birth.

Principal Components Analysis

Maternal vaginal microbiota

Following data transformation and cluster analysis, we applied Principal Components Analysis on maternal samples. The first 3 Principal Components (PC) retained 42.19% of the variance and were not possible to see differences between Clusters 1 and 2. We investigated lower PCs and found that these Clusters differentiate on the sixth PC, which retained 6.19% of the variance. Also, the top 10 contributing OTUs for variation of each PC pair is shown as arrows.

```
library(factoextra)
do=as.data.frame(otu_table(inputm.clr))
tax=as.data.frame(tax_table(inputm.clr))
rownames(do)=tax$LastRank
pca=prcomp(t(do))
map=as.data.frame(sample_data(inputm.clr))
c.kmeansM1=fviz_pca_biplot(pca, palette = "jco",label = "var",pointsize=2, repel=T,
                           title = "", select.var=list(contrib=10),
                           habillage = map$Clusters, addEllipses = T,
```

```
                            ellipse.level=.70, axes = c(1,2))
c.kmeansM2=fviz_pca_biplot(pca, palette = "jco",label = "var",pointsize=2, repel=T,
                            title = "", select.var=list(contrib=10),
                            habillage = map$Clusters, addEllipses = T,
                            ellipse.level=.70, axes = c(3,2))
c.kmeansM3=fviz_pca_biplot(pca, palette = "jco",label = "var",pointsize=2, repel=T,
                            title = "", select.var=list(contrib=10),
                            habillage = map$Clusters, addEllipses = T,
                            ellipse.level=.70, axes = c(1,3))
c.kmeansM4=fviz_pca_biplot(pca, palette = "jco",label = "var",pointsize=2, repel=T,
                            title = "", select.var=list(contrib=10),
                            habillage = map$Clusters, addEllipses = T,
                            ellipse.level=.70, axes = c(1,6))
ggarrange(c.kmeansM1, c.kmeansM2, c.kmeansM3, c.kmeansM4,common.legend = T,
          labels = "AUTO")
```

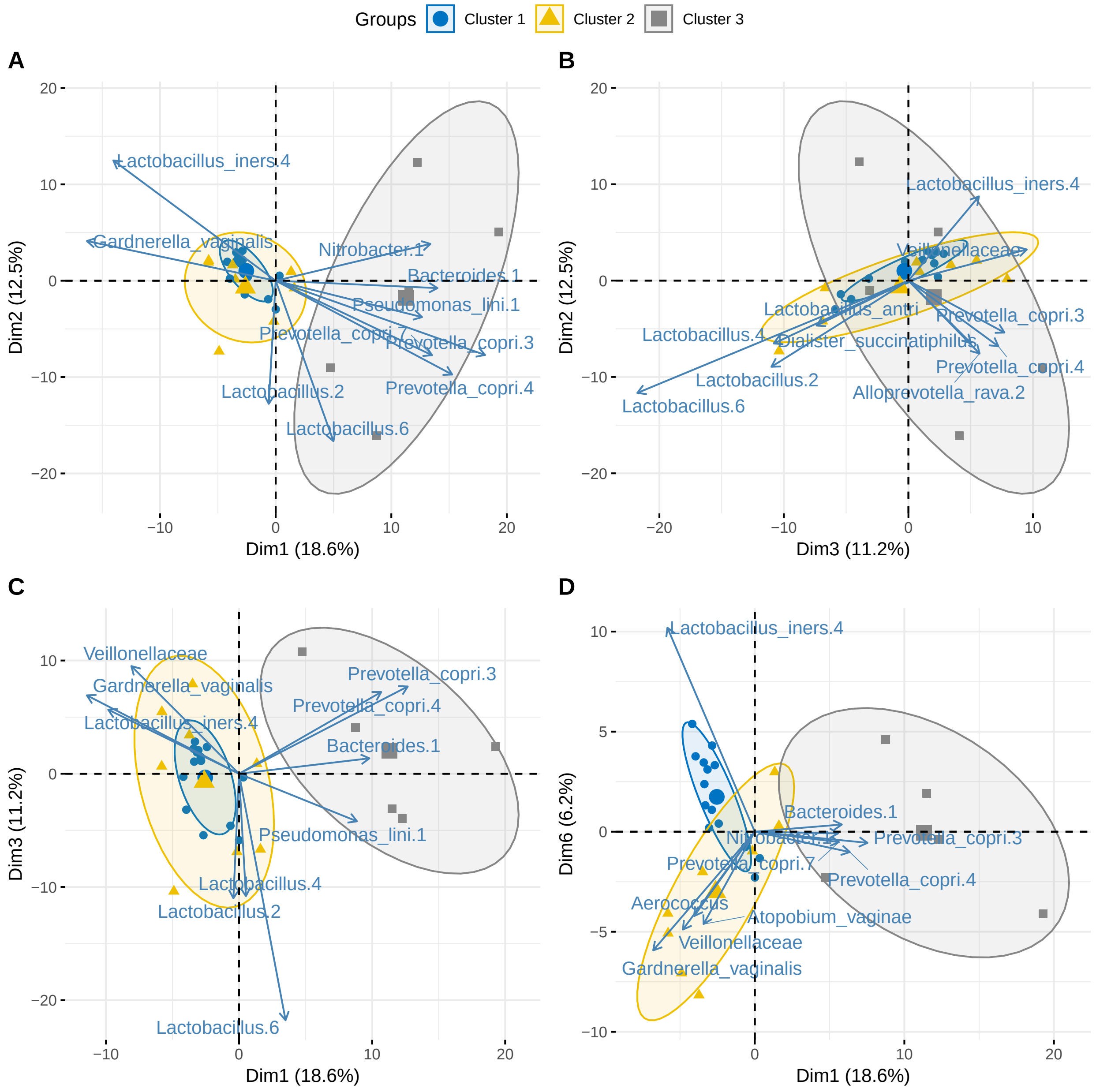

Figure 2: PCA of maternal vaginal microbiota and the top 10 contributing OTUs for variation: A) Principal component (PC) 1 and 2; B) PCs 2 and 3; C) PCs 1 and 3; D) PCs 1 and 6. Arrows represent the top 10 contributing OTUs for variation of each PC pair.

PCA of maternal samples was followed by an analysis of variance (PERMANOVA) on Aitchison distance, Euclidean distance of clr transformed data.

```
library(vegan)
df = as(sample_data(inputm.clr), "data.frame")
d = distance(inputm.clr, "euclidean")
input_adonis = adonis(d ~ Cluster, df)
kable(input_adonis$aov.tab) %>% kable_styling()
```

	Df	SumsOfSqs	MeanSqs	F.Model	R2	Pr(>F)
Cluster	2	1139.433	569.7167	3.255113	0.2133785	0.001
Residuals	24	4200.531	175.0221	NA	0.7866215	NA
Total	26	5339.964	NA	NA	1.0000000	NA

Infants meconium microbiota

Following data transformation and grouping infants' samples according with maternal vaginal communities, we applied Principal Components Analysis. The first 2 PCs retained 30.06% of the variance and it was not possible to see differences. Also, the top 10 contributing OTUs for variation is shown as arrows.

```
doN=as.data.frame(otu_table(rn.clr))
tax=as.data.frame(tax_table(rn.clr))
rownames(doN)=make.unique(as.character(tax$LastRank))
pca=prcomp(t(doN))
map=as.data.frame(sample_data(rn.clr))
c.kmeansN=fviz_pca_biplot(pca, palette = "jco",label = "var",pointsize=2, repel=T,
                          title = "", select.var=list(contrib=10),
                          habillage = map$ClusterM, addEllipses = T,
                          ellipse.level=.70)
c.kmeansN
```

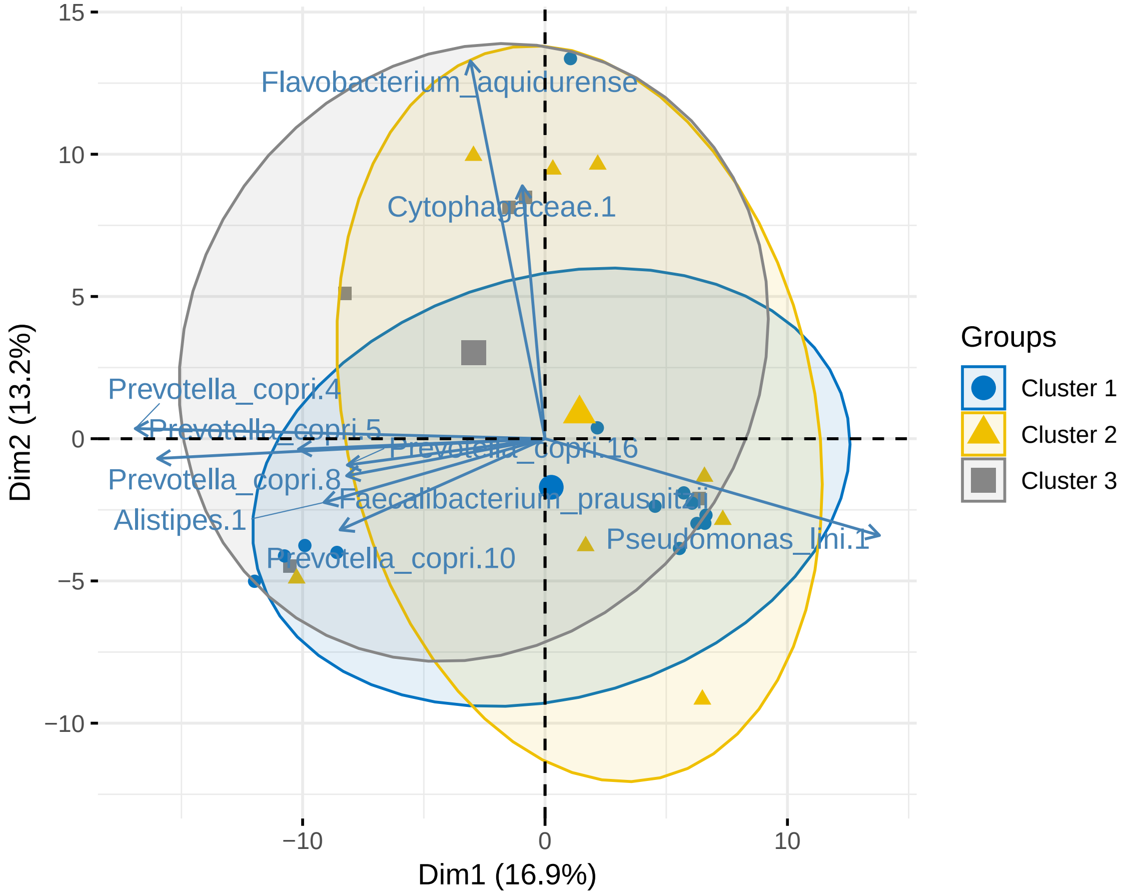

Figure 3: PCA of newborns gut's microbiome at birth. Arrows represent the top 10 contributing OTUs for variation of each PC pair.

PCA of infants samples was followed by an analysis of variance (PERMANOVA) on Aitchison distance, Euclidean distance on clr transformed data.

```
library(vegan)
df_newborns = as(sample_data(rn.clr), "data.frame")
d_newborns = distance(rn.clr, "euclidean")
input_adonis_n = adonis(d_newborns ~ ClusterM, df_newborns)
kable(input_adonis_n$aov.tab) %>% kable_styling()
```

	Df	SumsOfSqs	MeanSqs	F.Model	R2	Pr(>F)
ClusterM	2	617.4507	308.7253	1.129868	0.08946	0.207
Residuals	23	6284.5223	273.2401	NA	0.91054	NA
Total	25	6901.9729	NA	NA	1.00000	NA

Microbiome composition

```
mothers = names(sort(taxa_sums(inputm.clr), TRUE)[1:30])
top30mothers = prune_taxa(mothers, inputm.clr)
```

```r
map=as(sample_data(top30mothers),"data.frame")
otu=otu_table(top30mothers)
taxs=as.data.frame(tax_table(top30mothers))
#name the OTUs according to the toxonomy (keeping individual names)
rownames(otu)=taxs$LastRank
#put toghether counts and metadata
otu.map=data.frame(t(otu),map)
otu.map=rownames_to_column(otu.map,var = "SampleID")
#plotting
myplots=list()
top30=sort(as.factor(taxs$LastRank))
for (i in top30) {
  p1=ggboxplot(otu.map, x="Cluster", y=as.character(i), color = "Cluster",
               palette = "jco")+ xlim("Cluster 1", "Cluster 2", "Cluster 3")+
    xlab("")+ theme(axis.text.x = element_text(size = 9, hjust = .1, angle = -45),
                    axis.text.y = element_text(size = 9))+
    stat_compare_means(label = "p.format")
  myplots[[i]]<-p1
}
ggarrange(plotlist = myplots, common.legend = T) %>%
  annotate_figure(left = text_grob("Relative abundance (Centered log ratio)",
                                   rot = 90))
```

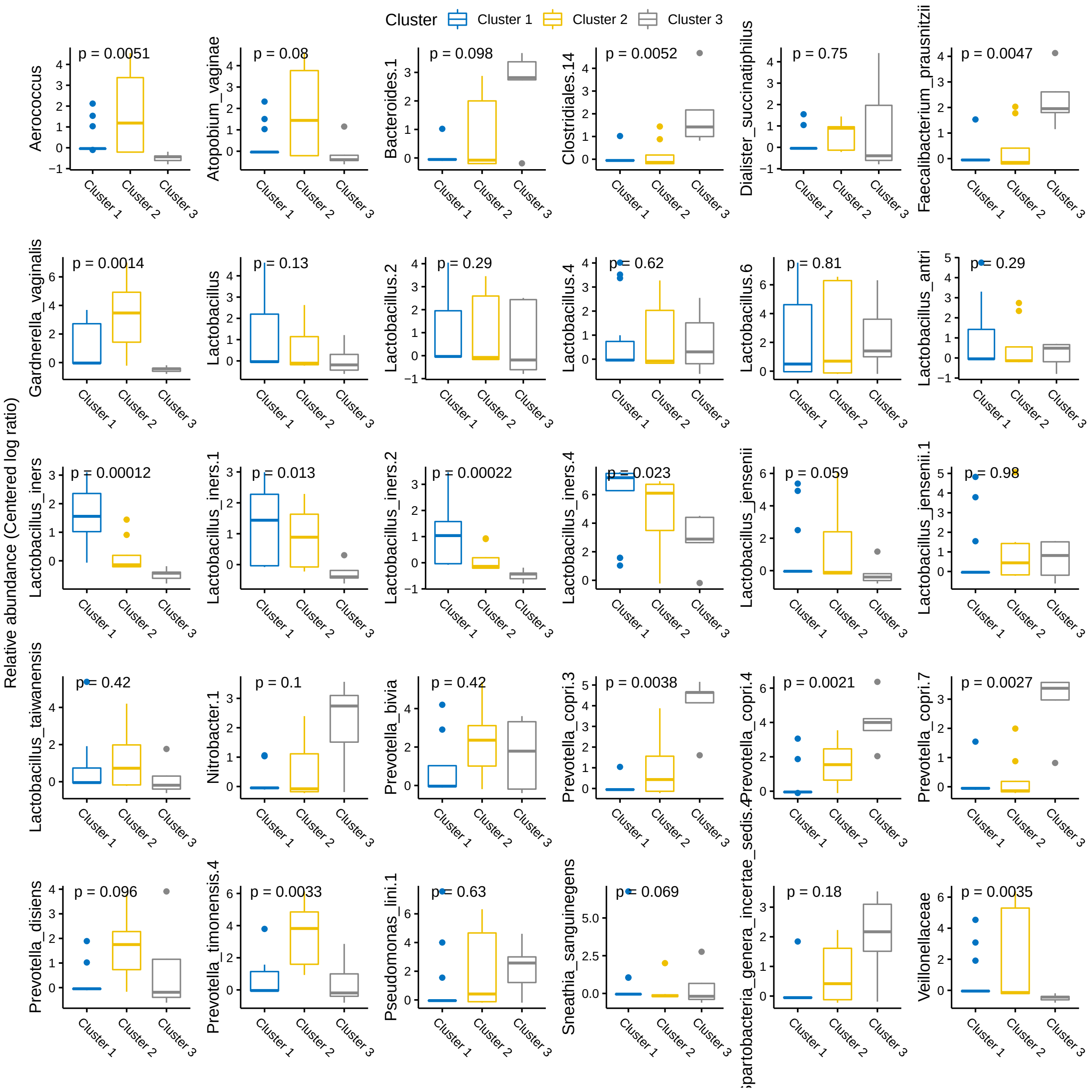

Figure 4: Top 30 OTUs from mother's vaginal microbial communities

```
babies = names(sort(taxa_sums(rn.clr), TRUE)[1:30])
top30rn = prune_taxa(babies, rn.clr)
map=as(sample_data(top30rn),"data.frame")
otu=otu_table(top30rn)
taxs=as.data.frame(tax_table(top30rn))
#name the OTUs according to the toxonomy (keeping individual names)
rownames(otu)=taxs$LastRank
#put toghether counts and metadata
otu.map2=data.frame(t(otu),ClusterM=map$ClusterM)
otu.map2=rownames_to_column(otu.map2,var = "SampleID")
```

```r
#ploting
top30=sort(as.factor(taxs$LastRank))
myplots=list()
for (i in top30) {
  p1=ggboxplot(otu.map2, x="ClusterM", y=as.character(i), color = "ClusterM",
               palette = "jco")+ xlim("Cluster 1", "Cluster 2", "Cluster 3")+
    xlab("")+font("ylab", size = 10)+
    theme(axis.text.x = element_text(size = 9, hjust = .1, angle = -45),
          axis.text.y = element_text(size = 9))+
    stat_compare_means(label = "p.format")
  myplots[[i]]<-p1
}
ggarrange(plotlist = myplots, common.legend = T) %>%
  annotate_figure(left = text_grob("Relative abundance (Centered log ratio)", rot = 90))
```

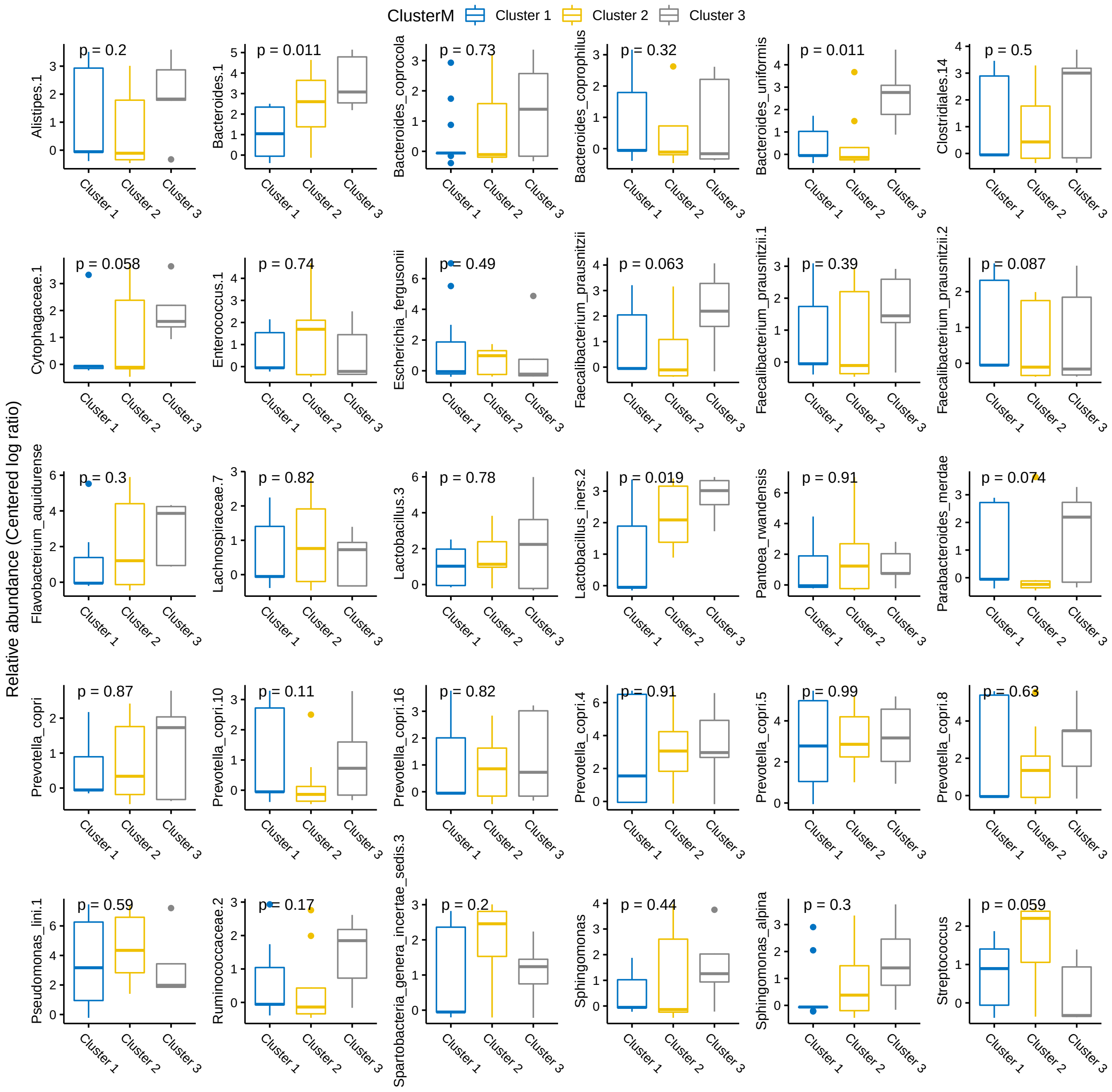

Figure 5: Top 30 OTUs from newborn's gut microbial communities
